# An alternative method to differentiate pleural effusion after leakage of artificial enteral nutrition formula

**DOI:** 10.1007/s12024-025-00982-0

**Published:** 2025-03-21

**Authors:** Fabian Picht, Karen Blümke-Anbau, Carolin Richter, Marco Weber

**Affiliations:** Institute of Legal Medicine, University of Halle-Wittenberg, Franzosenweg 1, 06112 Halle (Saale), Germany

**Keywords:** Autopsy, Medical malpractice, Pleural effusion, Chylothorax, Artificial enteral nutrition, Tricaprylin, 10.190: Autopsy, 10.020: Medicine, 10.010: Pathology, 10.040: Toxicology

## Abstract

**Purpose:**

This case report highlights a rare but fatal complication of artificial enteral nutrition due to feeding tube misplacement, leading to pleural effusion and respiratory failure. The primary objective was to determine whether enteral nutrition formula leakage contributed to the patient’s death and to differentiate the pleural effusion from other possible etiologies, such as chylothorax.

**Methods:**

A 59-year-old male patient with severe lung disease underwent left-sided partial lung resection and subsequently received enteral nutrition via a nasogastric feeding tube. After suspected feeding tube misplacement and formula leakage into the pleural cavity, autopsy and histological examination were performed. Cytological and chemical analyses, including gas chromatography-mass spectrometry, were employed to identify nutritional components in pleural effusion and to confirm the presence of enteral nutrition formula.

**Results:**

The autopsy revealed milky, turbid pleural effusion, aspiration pneumonia, and a rupture of the right visceral pleura. Cytological analysis confirmed granulocytic pleural empyema with rod-shaped Congo red-positive material resembling the enteral nutrition formula. Gas chromatography-mass spectrometry identified tricaprylin, a unique marker for enteral nutrition formula, confirming nutritional leakage into the pleural cavity.

**Conclusions:**

This case study emphasizes the necessity for radiological confirmation of feeding tube placement and the implementation of comprehensive diagnostic protocols for suspected cases of nutritional fluid leakage. Gas chromatography-mass spectrometry proved invaluable in distinguishing nutritional effusions from other potential etiologies by enabling the specific identification of enteral formula components. The high specificity and adaptability of gas chromatography-mass spectrometry render it an essential tool for forensic investigations and clinical diagnostics involving complex fluid analyses, facilitating evidence-based conclusions in critical care and postmortem contexts.

## Introduction

Artificial enteral nutrition (EN) is an essential form of therapy to support patients who are unable to meet their nutritional requirements through oral food intake. Compared with parenteral nutrition, this method utilizes the natural digestive tract, which offers numerous advantages in terms of maintaining intestinal integrity and avoiding complications [1].

The indications for EN are diverse and range from neurological diseases that lead to dysphagia to gastrointestinal disorders and oncological diseases in which the energy and nutrient requirements are increased. EN is a critical component of intensive care medicine, with the potential to improve the outcomes of critically ill patients [2]. It can be administered through various access routes, including nasogastric tubes, percutaneous endoscopic gastrostomy tubes or jejunal access.

Despite its benefits, EN is associated with several potential complications. These include, but are not limited to, aspiration pneumonia, gastrointestinal perforation, mechanical tube displacement, and metabolic disturbances, such as refeeding syndrome [3]. One of the most concerning complications is the malpositioning of the feeding tube (FT), which may result in the inadvertent infusion of nutritional content into non-gastrointestinal spaces such as the lungs or pleural cavity, leading to severe respiratory or systemic complications [4].

In addition to the benefits, EN also poses challenges and risks, such as the risk of aspiration, mechanical complications or the development of refeeding syndrome. Consequently, its use requires careful monitoring and adaptation to the individual needs of the patient [3, 5, 6].

This case report describes a patient whose cause of death was suspected to be the result of the misplacement of a feeding tube. Autopsy, performed on behalf of the local prosecutor, revealed chyle-like milky pleural effusion, suggesting misplacement of the FT and subsequent feeding. The challenge was to identify the pleural effusion to verify the presence of the EN formula and to distinguish it from, for example, chyle. A recent case report suggested the detection of parenteral nutrition formula by measuring elevated levels of triglycerides, glucose, and potassium, which allows indirect conclusions about the presence of parenteral nutrition formula and the ability to differentiate it from pleural effusions of other origins [7]. Here, a unique methodological approach, direct and specific for differentiating between EN formula and similar effusion fluids, was performed.

## Case

### Clinical history

A 59-year-old patient was admitted to the hospital with a diagnosis of lung emphysema Gold IV/D, accompanied by recurrent spontaneous pneumothoraces. Consequently, a left-sided partial lung resection was performed with subsequent intensive care. The postoperative diet was created with EN formula administered via a nasogastric FT. The FT position control was realized by auscultation prior to the administration of EN formula (Nutrison Energy multifibre^®^ tube feed (Nutricia, Germany)) via the FT. The following morning, as part of a routine radiological examination of the thorax, a misplacement of the FT into the right lung was suspected, indicating that EN formula had been administered into the right lung. Accordingly, the tube position was corrected. However, the patient died later that day.

Other specified diagnoses were diabetes mellitus, type II after ethyl toxic pancreatitis and hypertension.

### Autopsy and histology

**Fig. 1:**
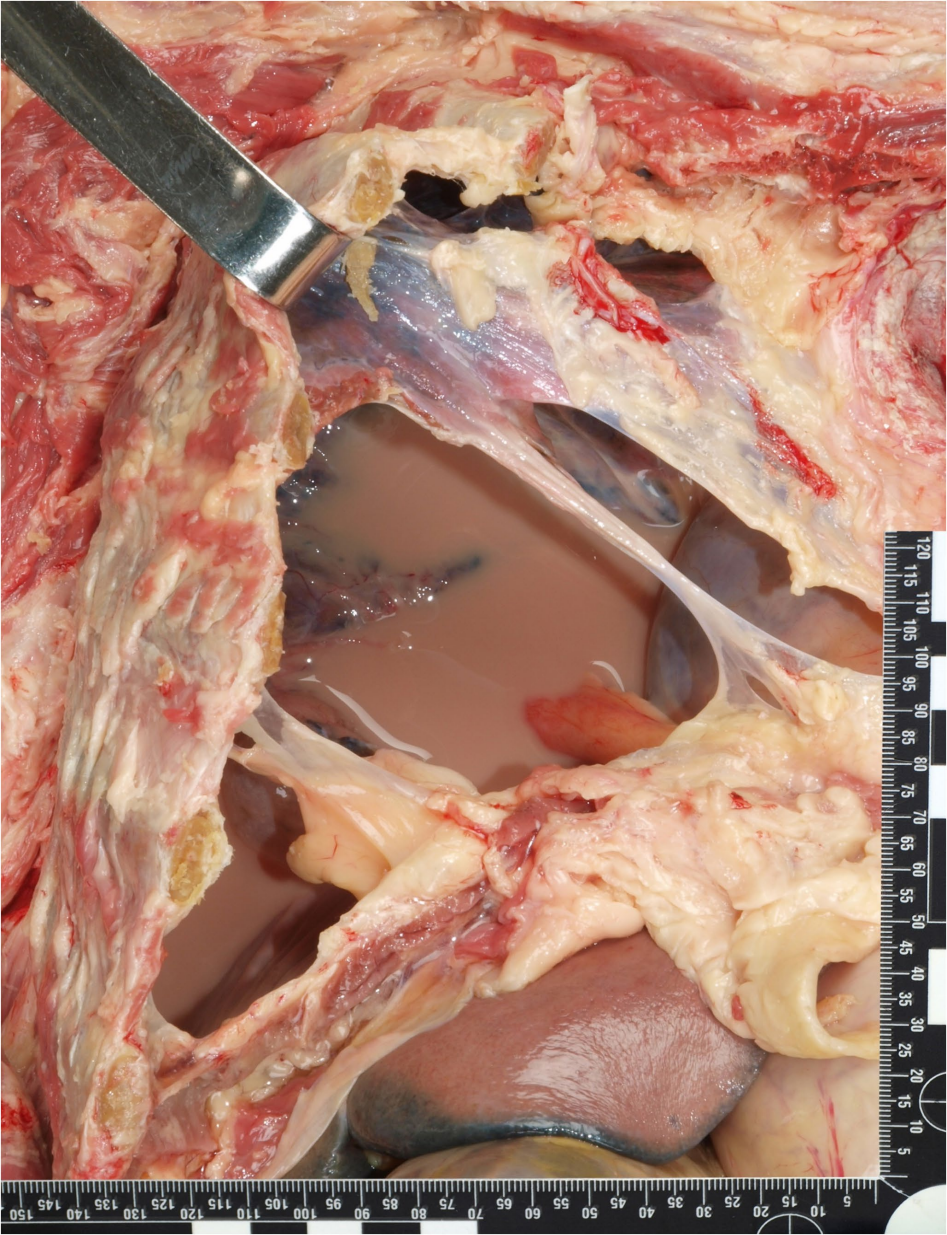
Autoptic findings of turbid, milky-brown liquid in the right chest cavity

**Fig. 2:**
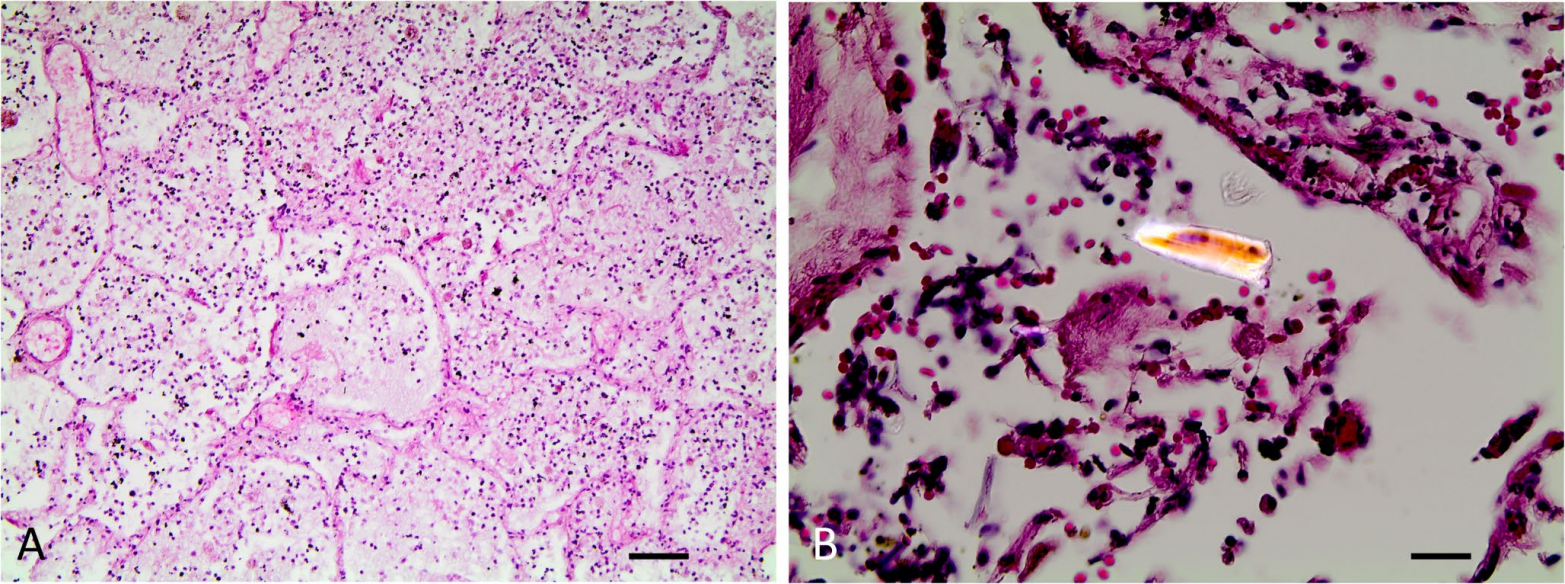
Histological detection of aspiration pneumonia via hematoxylin‒eosin staining of lung tissue (**A**) Overview (scale bar 100 μm), (**B**) closer view (scale bar 25 μm) with a foreign body in polarized light

The aim of the autopsy was to determine whether the leakage of EN formula into the pleural cavity contributed directly to the patient’s death. The autopsy findings revealed bullae consistent with chronic pulmonary emphysema. The surgery was performed without any complications. However, 350 ml of a turbid, milky-brown liquid was detected in the right thFig. (Fig. 1). The airways contained yellowish-brown mucus. In addition, the visceral pleura of the right lung was ruptured in certain areas. The lung tissue displayed a lack of ventilation and was rich in fluids. There was histological evidence of aspiration pneumFig. (Fig. 2). The hypothesis of prolonged oxygen deficiency was substantiated by the histology of the central nervous system, which revealed elective necrosis of nerve cells. Furthermore, corroborative findings included signs of chronic kidney disease, chronic heart insufficiency and coronary disease, as well as signs of the specified arterial hypertension.

To elucidate the nature of the liquid in the right thorax, a combination of cytological and chemical-toxicological investigations was performed.

### Cytological analysis

For comparison, a specimen of the EN formula Nutrison Energy multifibre^®^ tube feed (Nutricia, Erlangen, Germany) was obtained. Following centrifugation, smears of the EN formula and the pleural effusion from the right thoracic cavity were prepared. The preparations were subsequently stained via different methods, including hematoxylin‒eosin and Congo red (all chemicals were purchased from Carl Roth, Germany).

**Fig. 3:**
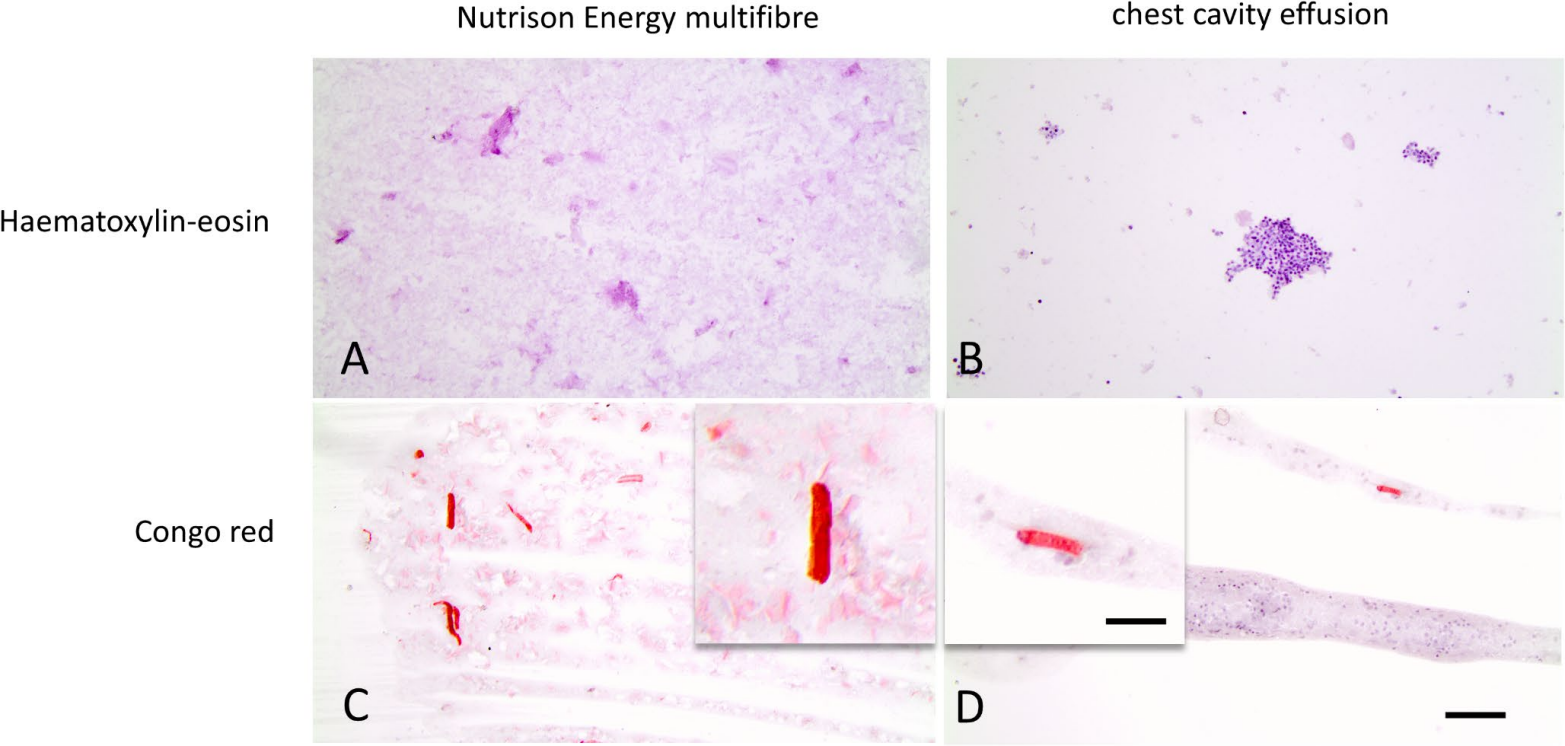
Comparison of smears from Nutrison Energy multifibre tube feed (**A** and **C**) and pleural empyema from the right chest cavity (**B** and **D**) stained with hematoxylin‒eosin (upper row) and Congo red (lower row), scale bar 25 μm. Note that the Congo red-stained smears of both the control and pleural empyema samples contained similar rod-shaped Congo red-positive particles (inserts in **C** and **D**, scale bar 12,5 μm)

Numerous granulocytes, predominantly exhibiting a lobed morphology, were found in the right thoracic cavity smear, confirming the diagnosis of pleural empyema. In addition, Congo red-positive material was detected in both the control smear from the EN formula and the pleural effusion smear. The proportions of EN in the chest cavity may be represented by rod-shaped Congo red-positive material (Fig. 3).

### Chemical analysis

#### Chemicals and reagents

Papaverine at a concentration of 0.1 mg/mL was obtained from Cerilliant Corporation (Round Rock, Texas, USA) and was used as the internal standard. Normocaloric tube feeding solution was purchased from Nutricia (Erlangen, Germany). Analytical-grade methanol was obtained from Merck (Darmstadt, Germany).

#### Sample Preparation

*A* 20 µL sample was introduced into a 2 mL reaction vial, spiked with 20 µL of papaverine and mixed with 960 µL of methanol. The mixture was vortexed for 30 s and centrifuged (5 min, 14000 rpm). Finally, 250 µL of the supernatant was transferred to a 1.5 mL glass vial for analysis.

#### Gas chromatography–mass spectrometry

Gas chromatography–mass spectrometry (GC/MS) analysis was performed via an Agilent 7890 gas chromatograph equipped with a 5975 C mass selective detector and an HP-5MS capillary column (30 m × 0.2 mm I.D., 0.25 μm film thickness) (Waldbronn, Germany). The injector was operated in splitless mode with an injection volume of 1 µL. The inlet temperature and helium flow rate were set to 280 °C and 1 mL/min, respectively. The oven temperature gradient program was as follows: hold an initial temperature of 160 °C for 1 min, ramp to 180 °C at 10 °C/min, then ramp to 220 °C at 5 °C/min, ramp to 270 °C at 15 °C/min, ramp to 300 °C at 10 °C/min, and finally hold for 5 min. The source and transfer line temperatures were 280 °C and 300 °C. The mass spectrometer was run in positive electron ionization mode at 70 eV. The acquisition range was set to 50–500 m/z in scan mode.

### Analysis of the samples

**Fig. 4:**
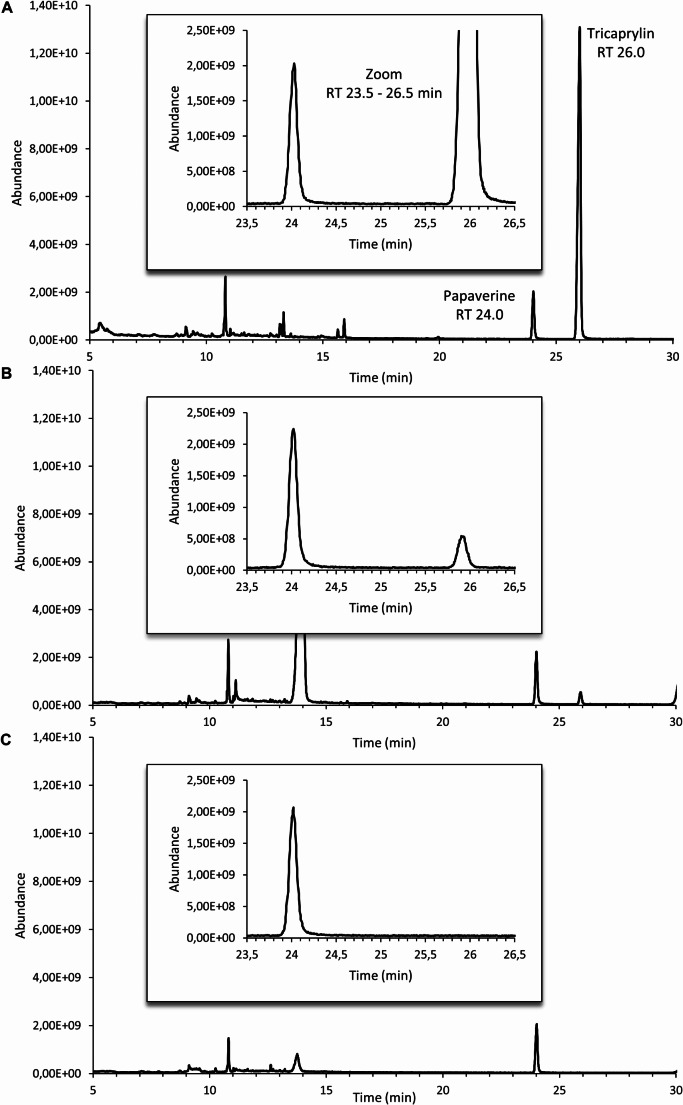
GC/MS chromatograms including a magnified view of the relevant retention time area: **a**) Nutrison energy multifibre tube feed; **b**) case sample; **c**) representative negative sample

Assuming that the pleural effusion from the present case was contaminated with the EN formula, a sample, the EN formula itself and one pleural effusion sample where EN could be excluded (negative sample) were analyzed. The resulting chromatograms were checked visually for characteristic differences. At 26 min, a signal appeared, which was exclusively found in the EN formula and in the case sample but not in the negative samples (Fig. 4).

**Fig. 5:**
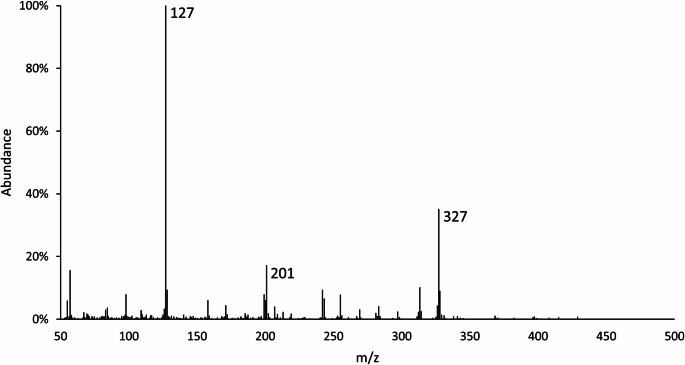
Mass spectrum of tricaprylin

Both peaks were identified as tricaprylin (1,2,3-propantriyl-trioctanoat) by using NIST 98 library [8], with quality scores of 91 and 87 (Fig. 5). The negative sample was analyzed for the presence of tricaprylin, and the results obtained indicated the absence of the analyte, thereby confirming that the circulating tricaprylin had not migrated from the blood into the thoracic effusion.

According to the manufacturer of the tube feed solution, the solution is composed of carbohydrates, fiber, protein, salt, various vitamins and lipids [9]. The latter also includes medium-chain triglycerides (MCTs) at a concentration of 9.2 mg/mL. One of these MCTs is tricaprylin, which is an organic chemical compound in which three hydroxyl groups of glycerol are esterified by octanoic acid. MCTs such as tricaprylin are fatty acids that can be obtained from coconut or palm kernel oil [10].

## Discussion

This case highlights a rare but severe complication of enteral nutrition, involving the misplacement of a FT and the subsequent leakage of EN formula into the pleural cavity. The clinical course underscores the potential for fatal outcomes even when the tube misposition is identified and corrected promptly.

### Clinical implications of FT malposition

FT misplacement is a well-recognized complication of enteral nutrition and has been associated with severe respiratory and systemic outcomes, including pleural effusion and aspiration pneumonia. In a reported case, a 61-year-old female experienced “nutrothorax” due to a misplaced nasogastric feeding tube, leading to severe complications such as aspiration pneumonia and pleural effusion [11]. Similarly, another case highlighted iatrogenic empyema resulting from the malposition of a nasogastric tube into the bronchial tree, underscoring the potential for life-threatening pulmonary complications [6].

In the present case, the initial auscultation method used to verify FT position proved insufficient, leading to intrabronchial misplacement and EN formula administration into the right lung. Studies have shown that auscultation is an unreliable method for confirming nasogastric tube placement [12]. Radiologic confirmation remains the gold standard for verifying FT placement, despite some drawbacks such as possible misinterpretation, feeding delay, X-ray exposure, and costs [13]. The findings of the current case study reinforce the critical importance of radiological confirmation for FT positioning, especially in the context of critically ill patients, where complications can rapidly escalate.

### Diagnostic challenges

The chyle-like appearance of the pleural effusion initially suggested a differential diagnosis of chylothorax. Chyle leaks can be the result of many different causes [14, 15]. Chylothorax can also be a rare complication of pancreatitis [16]. However, cytological and chemical analyses, including GC/MS, confirmed the presence of EN formula components, specifically tricaprylin, an MCT. In principle, tricaprylin represents a suitable candidate for the differentiation of endogenous effusions if EN leakage is suspected. Unlike long-chain triglycerides, which enter the blood through the lymphatic system, MCTs are presumed to be absorbed directly into the portal circulation [12]. This suggests that the presence of tricaprylin in endogenous effusions is minimal or undetectable. Lipoprotein electrophoresis has been suggested to be the gold standard for detecting ascites and pleural effusions. However, the detection of (total) triglyceride levels has been mentioned as an adequate alternative for the detection of chyle [13]. This method is not widely available and is associated with higher costs. The identification of tricaprylin was critical in establishing the causal link between the FT misplacement and the pleural effusion.

GC/MS proved to be a valuable tool in differentiating nutritional formula leakage from endogenous pleural effusion. Tricaprylin was absent in the negative control sample, further supporting the conclusion that its presence was due to EN formula contamination rather than endogenous lipid metabolism.

### Pathophysiological insights

The detection of EN formula in the pleural cavity is a critical finding, as it can lead to significant respiratory complications. Aspiration of EN formula into the lungs can result in chemical pneumonitis and increase the risk of developing aspiration pneumonia [17]. The presence of foreign material in the pleural space can also contribute to inflammation and infection, exacerbating respiratory distress [18]. In this case, the histological findings confirmed the development of aspiration pneumonia, likely due to the introduction of EN formula into the pulmonary system.

The accumulation of turbid, milky fluid in the pleural cavity, as observed in this patient, can impair lung expansion and gas exchange, leading to atelectasis and respiratory compromise [17]. The rupture of the visceral pleura further complicates the clinical picture, as it allows the translocation of fluids into the pleural space, increasing the risk of pleural effusion and subsequent respiratory failure [7, 18]. These pathological changes can create a cycle of deteriorating respiratory function, ultimately contributing to the patient’s demise.

This case underscores the importance of meticulous verification of feeding tube placement and the need for prompt recognition and management of complications arising from EN administration [13]. Implementing rigorous protocols for tube placement verification and monitoring can help prevent such adverse outcomes.

### Study limitations and future directions

This case report is limited by its retrospective nature and the lack of quantitative tricaprylin measurements. Future studies should explore the broader application of GC/MS in diagnosing nutritional agent leakage and establishing reference values for EN formula components in pleural effusions. Additionally, clinical guidelines should emphasize the routine use of imaging techniques for FT placement verification to prevent similar adverse events.

## Conclusion

This case demonstrates the significance of accurate diagnostic techniques in identifying rare complications of enteral nutrition and highlights the relevance of GC/MS in differentiating pleural effusions caused by nutritional formula leakage. The method developed for the presented case is applicable to analogous cases and adaptable. To the best of the author’s knowledge, the presented methodological approach describes the first analytical method for the direct detection of an EN food ingredient in body fluids. The findings further reinforce the necessity for stringent FT placement protocols and comprehensive diagnostic approaches in critical care settings.

## Key points

1. This case highlights a fatal complication of enteral nutrition involving feeding tube misplacement, leading to pleural effusion, aspiration pneumonia, and respiratory failure.

2. The feeding via an incorrectly placed nasogastric feeding tube can lead to severe complications, including death, during medical treatment.

3. Macroscopic differentiation between artificial enteral nutrition formulas and similar cavity fluids can be challenging.

4. This case demonstrates the use of gas chromatography-mass spectrometry in conjunction with cytology to differentiate pleural effusions caused by enteral nutrition formula leakage and emphasizes its value in rare autoptic scenarios.

## Data Availability

The datasets generated during the current study are available from the corresponding author on reasonable request.

## Abbreviations

EN: Enteral nutrition
FT: Feeding tube
GC/MS: Gas chromatography–mass spectrometry
MCT: Medium-chain triglyceride
